# Acupressure for older people with cognitive impairment: a systematic review and meta-analysis of randomized controlled trials

**DOI:** 10.3389/fpsyt.2025.1548878

**Published:** 2025-04-25

**Authors:** Hongkun Zhang, Luwen Zhu, Minmin Wu, Wenjing Song, Jinting Li, Qiang Tang, Jiongliang Zhang

**Affiliations:** ^1^ Department of Tuina, The Second Affiliated Hospital of Heilongjiang University of Chinese Medicine, Harbin, China; ^2^ Rehabilitation Center, The Second Affiliated Hospital of Heilongjiang University of Chinese Medicine, Harbin, China; ^3^ Heilongjiang Provincial Key Laboratory of Brain Function and Neurorehabilitation, The Second Affiliated Hospital of Heilongjiang University of Chinese Medicine, Harbin, China; ^4^ Heilongjiang University of Chinese Medicine, Harbin, China

**Keywords:** cognitive impairment, acupressure, cognition, mood, elderly

## Abstract

**Introduction:**

Cognitive impairment (CI) is becoming more common in the older population (≥60 years old) and has become a burden and challenge in an aging society. Acupressure is a non-invasive, safe, and cost-effective modality in Chinese medicine. Its therapeutic effects are achieved by stimulating specific points to restore balance in the flow of qi along the meridians, thereby enhancing the physiological functions of body systems and organs. This study aimed to conduct a meta-analysis to evaluate the effects of acupressure on cognitive function, mood, and activities of living (ADL) in older adults with CI.

**Methods:**

A comprehensive database search was performed using PubMed, Embase, Cochrane Library, Web of Science, Sinomed, CNKI, Wanfang, and VIP databases to identify randomized controlled trials (RCTs) investigating the effects of acupressure versus non-acupressure in elderly patients with CI. We searched on June 6, 2024 for trials that met our predefined inclusion and exclusion criteria from database construction to the present. An additional search was conducted from June 6, 2024 to March 16, 2025. Data were extracted, the literature was reviewed, and the methodological quality of the included trials was assessed. A meta-analysis was performed using StataSE version 16.

**Results:**

The meta-analysis included 1,149 patients from 15 RCTs. Results showed that compared with the control group, cognition (mean difference [MD] = 2.36, 95% confidence interval = 1.71 to 3.00, P < 0.001, I² = 41.8%), agitation (MD = -1.51, 95% confidence interval = -2.52 to -0.50, P = 0.003, I² = 0%) and depression (standardized mean difference [SMD] = -1. 33, 95% confidence interval = -1.80 to -0.86, P < 0.001, I² = 32.2%) improved. However, no significant differences were observed in ADL.

**Conclusion:**

This systematic review provides valuable evidence for using acupressure to improve cognitive function and mood in older adults with CI. In the future, acupressure may improve cognition and mood in this demographic. More studies on acupressure are needed to generate stronger and more robust evidence.

**Systematic review registration:**

PROSPERO, identifier: CRD42024556579.

## 1 Introduction

As the population ages and life expectancy increases, cognitive impairment (CI) is an increasingly serious societal challenge (1). Cognitive decline, mild CI, and Alzheimer’s disease have become common among the older population, and the number of people with dementia with more severe CI is increasing (2, 3). By 2030, the number of people with dementia worldwide will reach 75 million and will almost double to 130 million by 2050 (1). Patients with CI often have abnormal pathological changes in the brain, and symptoms include cognitive dysfunction, mood changes, and considerable dysfunction in activities of daily living (ADL) (4, 5). The rapidly increasing number of people with CI poses a substantial social and economic burden (6). Therefore, finding an economical, effective, and safe non-drug treatment method is worthwhile.

Older people with CI may have other systemic diseases, and using certain medications can aggravate their CI and result in adverse effects (7). Studies have shown that traditional Chinese medicine may be a beneficial complementary, non-drug approach to improve and treat CI in the older population (8). Acupressure is a traditional Chinese medicine therapy that uses massage techniques on the body’s acupuncture points to dredge the meridians based on the principles of acupuncture. Acupressure can be performed using different fingers, knuckles, or appropriate tools and is a non-invasive treatment method (9). Several types of acupressure exist, including Shiatsu, Jin Shin Do, auricular acupressure, and Tapas acupressure techniques (10). Acupressure is similar in principle to acupuncture in that it produces certain physiological healing effects through the acupuncture points (11, 12). According to the Zang-Fu theory of traditional Chinese medicine, the heart governs the mind and emotions (13). The mechanism of action of acupressure is based on the core concept of Chinese medicine, Qi, which can be best described as the biological energy that sustains living things. Psychosomatic pathologies are explained by Qi disorders in the organs, meridians, or systems of the body, such as Qi deficiency or excess (14). CI is caused by visceral disorders, poor Qi movement, and emotional disorders (13). Acupressure can stimulate these points to correct imbalances in the flow of Qi through the meridians, achieving therapeutic benefits by improving the physiological functions of the body’s systems or organs (15). Similar to acupuncture, it is gradually showing advantages in some difficult-to-treat diseases (16, 17). Acupressure therapy produces a similar soreness and swelling sensation as acupuncture to produce a clinical effect known as “de qi” (18). Compared to acupuncture, acupressure is not invasive and therefore has a better safety profile (19).

A previous study has shown that acupressure can improve cognitive function in older people with CI, specifically in counting, classification, sustained response, and semantic fluency (20). In addition to this, a recent study has demonstrated that acupressure is effective in improving cognitive function in older adults with CI, mainly in the areas of working memory, executive function, and language skills (21). Acupressure has the potential to become a low-cost alternative treatment in the future, supported by evidence-based medicine. However, there is no evidence-based research on the effects of acupressure on older people with CI. The purpose of this systematic review and meta-analysis was to explore the effects of acupressure on cognitive function, mood, and ADL in older people with CI and to provide evidence for clinicians and rehabilitation therapists.

## 2 Materials and methods

The review was conducted and reported following the Preferred Reporting Items for Systematic Reviews and Meta-Analyses (PRISMA) guidelines, details of which can be found in **Appendix Table 1** in **Supplementary Material** (22). The study protocol (registration no. CRD42024556579) is registered in PROSPERO.

### 2.1 Search strategy

A systematic search of articles was performed using PubMed, Embase, Cochrane Library, Web of Science, Sinomed, CNKI, Wanfang, and VIP databases from inception to June 2, 2024. The additional searches were conducted from the first search to March 16, 2025. No restrictions were imposed on the language of the articles included in the search. The detailed search strategy is provided in **Appendix Table 2** in **Supplementary Material**.

### 2.2 Eligibility and exclusion criteria

The Populations, Interventions, Comparisons, Outcomes, and Study Types (PICOS) framework was used to develop inclusion criteria as follows: (a) populations: older people (≥60 years old) with mild cognitive impairment (MCI) or Alzheimer’s disease (AD); (b) interventions: acupressure (not limited to any acupoints in any part of the body); (c) comparisons: usual care, medication, health education, no intervention, or other non-acupressure interventions; (d) outcomes: cognition, mood, and ADL; and (e) study design: randomized controlled trials (RCTs).

The following conditions were used to exclude studies: (a) people under 60 years of age or with other chronic diseases; (b) experimental groups that included non-acupressure interventions; (c) studies that were not RCTs; and (d) letters and conference abstracts. After full-text reading, the exclusion criteria were applied. The titles of the excluded articles and the reasons for their exclusion are shown in **Appendix Table 3** in **Supplementary Material**.

### 2.3 Selection criteria

Two reviewers separately checked the titles and abstracts of the publications. If a disagreement arises, it is resolved through discussion with the third author, and a decision is reached collaboratively. For additional assessment, the full texts of the studies that could not be identified by title or abstract screening were read. The reference lists of the relevant publications were checked to ensure no relevant studies were missed.

### 2.4 Data extraction

Two reviewers extracted the following data from the experimental and control groups of the selected studies: authors, country, year of publication, age distribution, proportion of males, study design, sample size, population, cognitive function scores, intervention conditions (acupoints, frequency, and duration), and outcome measures.

### 2.5 Risk of bias assessment and certainty of evidence

The risk of bias was assessed using the Physiotherapy Evidence Database (PEDro) scale. On the 11-item PEDro scale, higher scores (lowest score = 0; highest score = 10) indicated better methodological quality. Studies were categorized according to their quality: excellent (9–10), good (6–8), fair (4–5), and poor (≤3) (23, 24).

The Grading of Recommendations Assessment, Development, and Evaluation (GRADE) guidelines were used to assess the certainty of evidence, which was divided into four levels: high, moderate, low, and very low. The degree of evidence depends on the risk of bias, inconsistency, imprecision, indirectness, and the potential for publication bias (25).

Two reviewers assessed the methodological quality and reliability of the evidence. In cases of disagreement, a third reviewer was consulted to make the final decision.

### 2.6 Data analysis

When outcomes were assessed using different rating instruments, they were summarized based on the change in mean standard deviation from before to after the intervention. For all other cases, post-intervention data were used for summarization. Data were combined using the mean difference (MD) for outcomes assessed on the same scale, whereas the standardized MD (SMD) was calculated for outcomes assessed using different scales. To assess statistical heterogeneity, the estimated I² values were used. The I² value of 0% to 40% indicates heterogeneity that is likely not significant, 30% to 60% suggests moderate heterogeneity, 50% to 90% reflects substantial heterogeneity, and 75% to 100% indicates considerable heterogeneity (26). For each outcome, random effects models were used (27). A P-value <0.05 was considered statistically significant for all comparisons. Sensitivity analysis was performed to assess the impact of single studies on the pooled results by excluding them individually. Publication bias was assessed using Egger’s regression test (28). If the data were sufficient, subgroup analyses were performed according to the intervention time. Data analysis and forest plot construction were performed using the StataSE software (version 16.0; StataCorp LP, College Station, TX, USA).

## 3 Results

### 3.1 Selected studies

**Figure 1:**
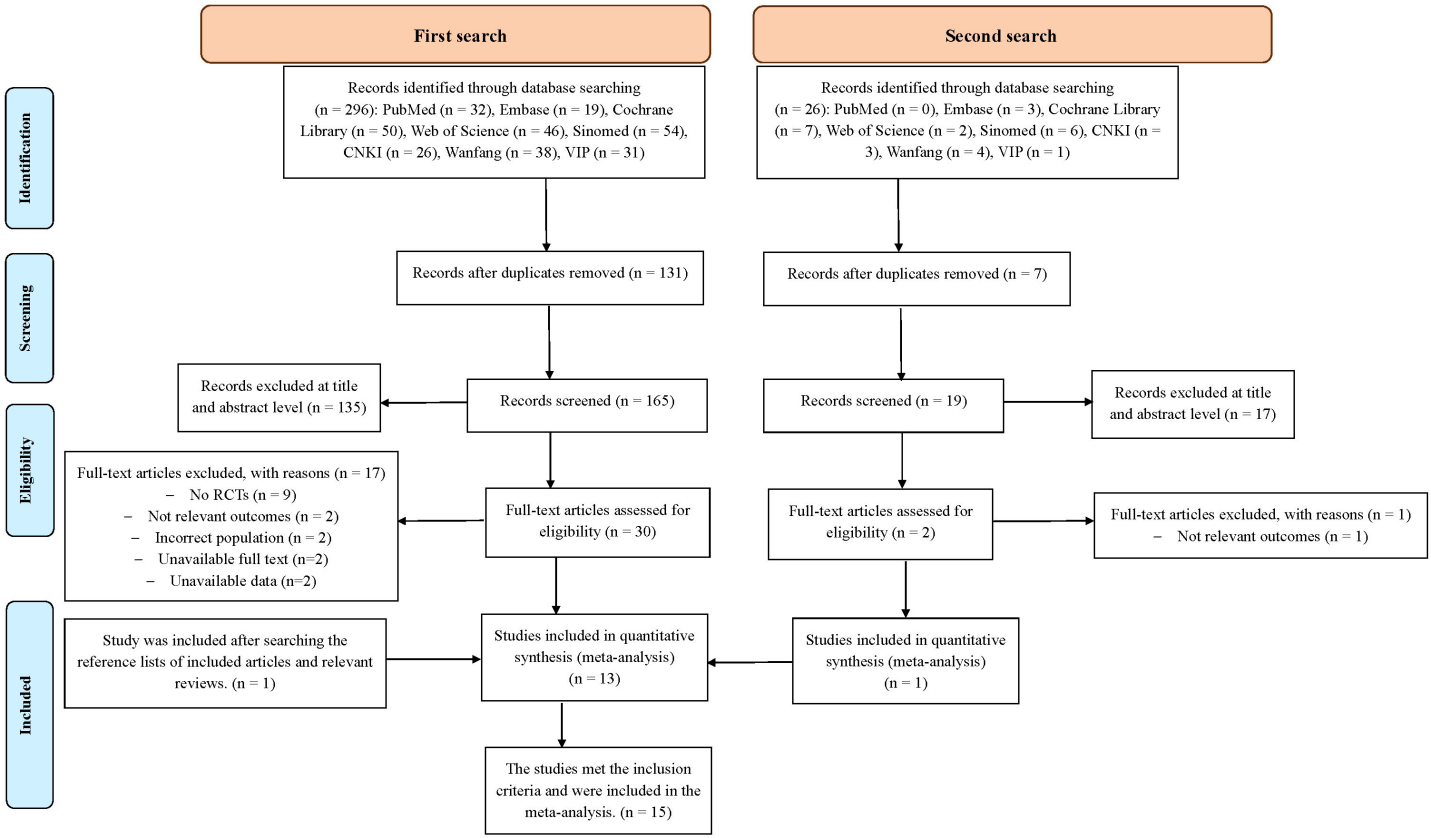
PRISMA flow chart for the study selection.

**Figure 1** illustrates the literature search and review process. We identified 296 articles, of which 131 were removed owing to duplication. In total, 135 articles were screened after reviewing their titles and abstracts. The full text of 30 potentially relevant studies was reviewed, and 15 articles met the inclusion criteria. After manually searching the reference lists, we identified one more relevant study; thus, 16 studies were included. However, two of the 16 studies did not provide sufficient data for the meta-analysis. Thus, 14 articles were included in the final analysis.

### 3.2 Characteristics of the included studies

The characteristics of the 14 included studies (20, 21, 29–41) can be obtained in **Table 1**. The language of the included literature was English. A total of 1,101 older people with CI were included in the 14 studies. The age range of the experimental and control groups was between 60 and 91 years old. The included studies were published between 2012 and 2024, of which seven were from China (29–34, 36, 41), three from Taiwan, China (20, 21, 38), one from Hong Kong, China (35), one from Vietnam (37), one from Italy (39), and one from Spain (40). Regarding the experimental groups in the included studies, 11 studies (20, 21, 29, 30, 32–37, 40) used acupressure alone, one study (39) used acupressure combined with exercise, two study (31, 41) used acupressure combined with cognitive training, and one study (38) used aromatherapy acupressure. Only two studies (31, 41) were superimposed effects of acupressure and other therapies, and the remaining combination therapy variables were acupressure. Of the 14 included trials, five control groups received usual care (21, 29–31, 35), five received health education (20, 33, 34, 36, 41), two received drug treatment (32, 37), one received exercise (39), one received aromatherapy (38), and one control group received no intervention (40). Each treatment in each study lasted 5 to 60 min, with 1 to 3 treatments per day. The total training duration in these studies ranged from 4 weeks to 10 months.

**Table 1:** Characteristics of included articles.

Study	Country	Design	Populations	Cognitive function score (E/C)	EG						CG					
					Sample size	Age (year)	Male (%)	Intervention	Acupoints	Time, Frequency	Sample size	Age (year)	Male (%)	Intervention	Time, Frequency	Outcomes
Guo et al., 2024 (41)	China	RCTs	MCI	MoCA:19.2/19.5	25	≥60	40%	Acupressure; Cognitive therapy	Baihui (GV20), Sishencong (EX-HN1), Fengchi (GB20), Jingming (BL1), Yongquan (KI1), Neiguan(PC6), Shenmen (HT7), Zusanli (ST36), Sanyinjiao (SP6), Yintang (EX-HN3), Sibai (ST2), Xuanzhong (GB39), Taixi (KI3)	60 minutes each time; 2 times a week, for 6 weeks	23	≥60	60%	Health education	Same as EG	Cognition
Vo et al., 2024 (37)	Vietnam	RCTs	AD	MMSE: 16.4/17.0	32	Median (IQR): 68 (61.5 to 78.3)	31.3	Acupressure	Shenmen (HT7), Neiguan (PC6), Baihui (GV20), Yintang (EX-HN3), and Fengchi (GB20)	5 min for each acupoint, 5 times per week, for 4 weeks	31	Median (IQR): 66.0 (62.0 to 74.0)	51.6	Drug (5 mg of donepezil and 1 mg of risperidone)	Once tablet per day, respectively	Mood (agitation)
Lin et al., 2023 (21)	China Taiwan	RCTs	MCI	NA	46	Mean age: 77.7	37.0	Acupressure	Baihui (GV20), Sishencong (EX-HN1), Shenting (GV24), Fengchi (GB20), Shuigou (GV26), Neiguan (PC6), Shenmen (HT7), and Zusanli (ST36)	Each acupoint was pressed for 3 min, once a day, 5 times a week, for a total of 12 weeks.	46	Mean age: 75.1	39.0	Usual care	12 weeks	Cognition
Liu et al., 2022 (32)	China	RCTs	MCI	MMSE: 22.6/22.3	40	Mean age: 68.3	57.5	Acupressure	Taiyang (EX-HN5), Baihui (GV20), Sishencong (EX-HN1), Shenting (GV24), Fengchi (GB20)	Each acupoint twice a day, 4 times with 8 beats each time, a total of 32 beats, 20 minutes, for 6 months	40	Mean age: 68.1	62.5	Drug (donepezil)	One tabley per day, the rest once a day	Cognition, ADL
Sun et al., 2021 (36)	China	RCTs	MCI	MMSE: 26.9/26.4	38	≥60	26.3	Acupressure	Baihui (GV20), Fengchi (GB20), Shenting (GV24), Sishencong (EXHN1) and Taiyang (EX-HN5)	85 seconds per acupoint, 3 times a day, 5 times a week, for 6 months	38	≥60	26.3	Health education	Once a month, 1 hour each time, for 6 months	Cognition
Lanza et al., 2018 (39)	Italy	RCTs	AD	MMSE: 16.9/17.5	6	Mean age: 77.0	0	Shiatsu; Motor activity	Customized for each patient by therapist	Shiatsu: once a week, 40 minutes each time, for 10 months; Motor activity: 1 hour and 15 minutes each time, 3 times a week for 10 months	6	Mean age: 80.0	33.3	Motor activity	1 hour and 15 minutes each time, 3 times a week for 10 months	Cognition, ADL, Mood (depression)
Xu. 2017 (29)	China	RCTs	AD	NA	25	Mean age: 78.6	56	Acupressure (Auricular acupuncture and massage)	Fengchi (GB20), Taiyang (EX-HN5), Baihui (GV20), Shenting (GV24)	5 minutes each time, 2 times a day, for 5 months	25	Mean age: 77.6	48	Usual care	5 months	ADL
Wang. 2017 (31)	China	RCTs	MCI	MMSE: 23.2/23.5	60	≥60	NA	Acupressure; Cognitive therapy	Taiyang (EX-HN5), Baihui (GV20), Sishencong (EX-HN1), Shenting (GV24), Fengchi (GB20)	Acupressure: press each acupoint 32 times, for a total of 4 cycles, 3 times a day, 5 times a week, for 6 months; Cognitive training: once a day for 6 months	60	≥60	NA	Usual care	Once a day for 6 months	Cognition
Kwan et al., 2017 (35)	China Hong Kong	RCTs	AD	MMSE: 7.4/5.6	39	Mean age: 86.9	20.5	Acupressure	Baihui (GV20), Shenman (HT7), Yingtang (EX-HN3), Fengchi (GB20), and Neiguan (PC6)	10 minutes each time, 2 times a day, 5 days a week, for 2 weeks	39	Mean age: 87.1	17.9	Usual care	2 weeks	Mood (agitation)
Wang et al., 2016 (33)	China	RCTs	MCI	MoCA<26	50	Mean age: 67.8	31.0	Acupressure	Baihui (GV20), Fengchi (GB20), Yintang (EX-HN3), Yuzhen (GB12), Yifeng (SJ17), Sibai (ST2), Weizhong (BL40), Yongquan (KI1), Foot sole at the pad of the big toe	1 time a day, 20 to 30 minutes each time, for 3 months	50	Mean age: 67.8	31.0	Health education	3 months	ADL
Miao. 2016 (30)	China	RCTs	AD	MMSE: 19.9/19.7	25	Mean age: 79.6	72.0	Acupressure (Auricular acupuncture and massage)	Taiyang (EX-HN5), Baihui (GV20), Sishencong (EX-HN1), Shenting (GV24), Fengchi (GB20), Shangxing (GV23)	5 minutes each time, 2 times a day, for 5 months	25	Mean age: 79.4	68.0	Usual care	5 months	Cognition, ADL
Yang et al., 2015 (38)	China Taiwan	RCTs	AD	NA	56	Mean age: 85.3	82.1	aroma-acupressure	Baihui (GV20), Fengchi (GB20), Shenmen (HT7), Neiguan (PC6), Sanyinjiao (SP6)	Less than 15 minutes each time, once a day, five days a week, for four weeks, follow-up after 3 weeks	73	Mean age: 81.6	75.4	Apply 2.5% lavender essential oil to 5 acupoints	Less than 15 minutes each time, once a day, five days a week, for four weeks	Mood (agitation)
Rodríguez-Mansilla et al., 2015 (40)	Spain	RCTs	AD	MMSE<20	40	Range: 67 to 91	22.6	Ear acupressure	Shenmen (HT7), 159.C Myorelaxant (peripheral inferior concha, close to the spleen and liver Chinese points), Xin (Heart)	3-month intervention, follow-up after 2 months	35	Range: 67 to 91	22.6	No intervention	NA	Mood (depression)
Zeng et al., 2012 (34)	China	RCTs	MCI	MMSE: 24.2/23.2	42	Mean age: 70.3	35.7	Acupressure	Taiyang (EX-HN5), Anmian (EX-HN22), Neiguan (PC6), Shenmen (HT7), Sanyinjiao (SP6)	30 minutes per session, 5 times a week, for 6 months	40	Mean age: 70.5	22.5	Health education	NA	Cognition, Sleep
Lin et al., 2009 (20)	China Taiwan	RCTs	AD	NA	42	Mean age: 80.9	64.3	Acupressure	Fengchi (GB20), Baihui (DU20), Shenmen (HE7), Neiguan (PC6), Sanyinjiao (SP6)	Press each acupoint for 2 minutes, once a day, 15 minutes each time, 6 days a week, for 4 weeks	52	Mean age: 80.9	73.1	Health education	15 min per day, 6 days per week, for 4 weeks	Mood (agitation)

EG, experimental group; CG, control group; RCTs, randomized controlled trials; MMSE, Mini-Mental State Examination; MoCA, Montreal Cognitive Assessment; MCI, mild cognitive impairment; AD, Alzheimer’s disease; ADL, activities of living; NA, Not applicable.

Data are shown as mean standard deviation where appropriate.

### 3.3 Methodological quality and certainty of evidence

Among the 15 included studies, the mean PEDro scale score was 5.9, with scores ranging from 4 to 8. Eight studies (20, 21, 35, 36, 38–41) were rated as good quality, and the other seven (29–34, 37) as fair quality. **Table 2** provides the PEDro scores of the included studies. Ratings using the GRADE methodology for all outcome measurements were inconsistent and ranged from low to very low certainty (**Appendix Table 4** in **Supplementary Material**).

**Table 2:** PEDro scores for the included studies.

Study	Items of PEDro scale											Total scores
	1	2	3	4	5	6	7	8	9	10	11	
Guo et al., 2024 (41)	Yes	1	1	1	1	0	1	0	0	1	1	7
VO et al., 2024 (37)	Yes	1	0	1	0	0	0	0	0	1	1	4
Lin et al., 2023 (21)	Yes	1	1	0	0	0	1	1	1	1	1	7
Liu et al., 2022 (32)	Yes	1	0	1	0	0	0	0	1	1	1	5
Sun et al., 2021 (36)	Yes	1	0	1	0	0	1	1	0	1	1	6
Lanza et al., 2018 (39)	Yes	1	1	0	1	0	0	0	1	1	1	6
Xu. 2017 (29)	Yes	1	0	1	0	0	0	0	1	1	1	5
Wang. 2017 (31)	Yes	1	0	1	0	0	0	0	1	1	1	5
Kwan et al., 2017 (35)	Yes	1	1	1	1	0	1	1	0	1	1	8
Wang et al., 2016 (33)	Yes	1	0	1	0	0	0	0	1	1	1	5
Miao. 2016 (30)	Yes	1	0	1	0	0	0	0	1	1	1	5
Yang et al., 2015 (38)	Yes	1	1	0	0	0	1	1	1	1	1	7
Rodríguez-Mansilla et al., 2015 (40)	Yes	1	1	0	1	0	1	1	0	1	1	7
Zeng et al., 2012 (34)	Yes	1	0	1	0	0	0	0	0	1	1	4
Lin et al., 2009 (20)	Yes	1	0	1	1	0	1	0	1	1	1	7

PEDro, Physiotherapy Evidence Database.

Items of the PEDro scale: 1 = specified eligibility criteria (yes/no); 2 = random allocation; 3 = concealed allocation; 4 = comparability at baseline;

5, blinded subjects; 6, blinded therapists; 7, blinded assessors; 8, sufficient follow-up; 9, intention-to-treat analysis 10 = comparison between groups; 11 = point estimates and variability. For terms 2–11:1, the corresponding criterion is satisfied; 0, the criterion is not satisfied.

### 3.4 Outcomes synthesis

Among the 15 studies, two or more included the cognition score, agitation score, depression function score, and ADL score. A detailed description of all the scales used in this study is provided in **Appendix Table 5** in **Supplementary Material**.

#### 3.4.1 Cognition

**Figure 2:**
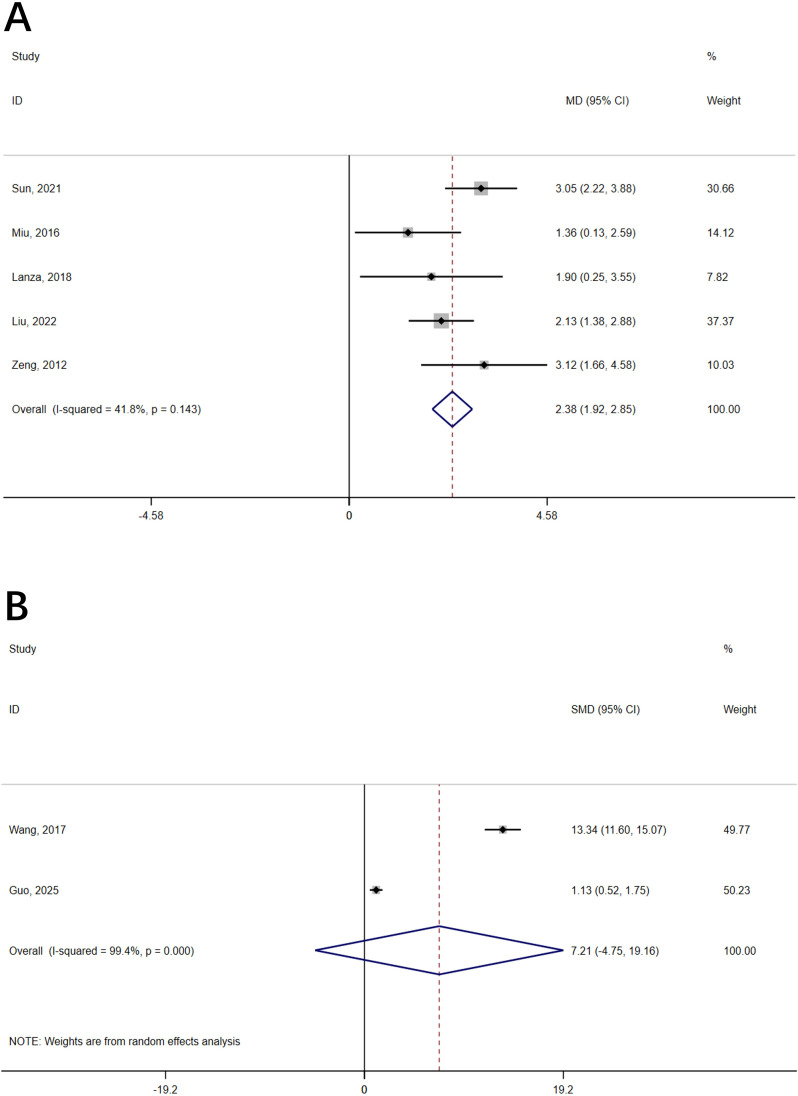
Forest plot of the effect of acupressure on cognition. **(A)** Acupressure alone; **(B)** Acupressure combined with cognitive therapy.

A total of seven studies (30–32, 34, 36, 39, 41) reported cognition scores. Five of these studies (30, 32, 34, 36, 39) in which acupressure was used as a control variable for the intervention were pooled and analyzed. A statistically significant difference was observed between the two groups (MD = 2.36, 95% confidence interval = 1.71 to 3.00, P<0.001, I² = 41.8%) (**Figure 2A**, **Table 3**), and there was no publication bias (P for Egger’s regression test = 0.77) (**Appendix Figure 1A** in **Supplementary Material**). The certainty of evidence for cognition was low. By excluding Sun (36) in the sensitivity analysis, heterogeneity was reduced by 31.7% (MD = 2.09, 95% confidence interval = 1.49 to 2.70, P = 0.043, I² = 10.1%) (**Appendix Figure 2A** in **Supplementary Material**). Subgroup analysis was performed according to the intervention time, and the patients were divided into three subgroups: 5 months (30), 6 months (32, 34, 36) and 10 months (39) (**Appendix Figure 3A** in **Supplementary Material**, **Table 3**).

**Table 3:** Results of the analysis of individual outcome indicators and their subgroups.

Outcomes	Groups		Number of comparisons	Total number of participants	Effect Size			Heterogeneity
					MD/SMD	95% confidence interval	P	I² (%)
Cognition	Overall Analysis		5	300	MD= 2.36	[1.71, 3.00]	<0.001	41.8
	Subgroup	5 months	1	50	MD= 1.36	[0.13, 2.59]	0.03	NA
		6 months	3	238	MD= 2.66	[1.98, 3.34]	<0.001	35.4
		10 months	1	12	MD= 1.90	[0.25, 3.55]	0.02	NA
Agitation	Overall Analysis		4	364	MD= -1.51	[-2.52, -0.50]	0.003	0
	Subgroup	2 to 3 weeks	2	207	MD= -1.43	[-2.81, -0.05]	0.04	0
		4 weeks	2	157	MD= -1.61	[-3.10, -0.13]	0.03	51.2
ADL	Overall Analysis		5	292	SMD= 0.67	[-0.11, 1.46]	0.09	89.2
	Subgroup	5 months	2	100	SMD= 0.88	[-1.54, 3.30]	0.48	96.6
		6 months	2	180	SMD= 0.33	[-0.33, 0.98]	0.33	79.4
		10 months	1	12	SMD= 1.17	[-0.11, 1.46]	0.07	NA

ADL, activities of living; MD, mean difference; SMD, standard mean difference; NA, Not appliable.

The control variable for the two included studies (31, 41) was acupressure combined with cognitive therapy. There was no statistically significant difference between the two groups(SMD = 7.21, 95% confidence interval = -4.75 to 19.16, P = 0.24, I² = 99.4%) (**Figure 2B**, **Table 3**).

#### 3.4.2 Agitation

**Figure 3:**
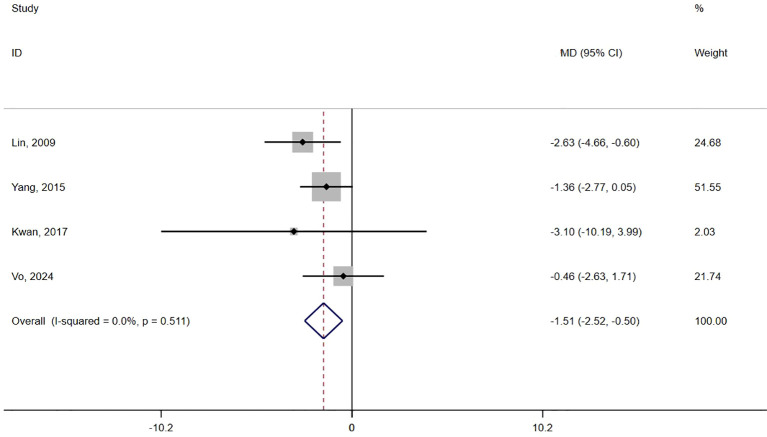
Forest plot of the effect of acupressure on agitation.

A total of four studies (20, 35, 37, 38) reported agitation scores. A statistically significant difference was observed between the two groups (MD = -1.51, 95% confidence interval = -2.52 to -0.50, P = 0.003, I² = 0%) (**Figure 3**, **Table 3**), and there was no publication bias (P for Egger’s regression test = 0.55) (**Appendix Figure 1B** in **Supplementary Material**). The certainty of evidence for the CMAI was low. Subgroup analysis was performed according to the intervention duration, with two subgroups: 2 to 3 weeks (35, 38) and 4 weeks (20, 37) (**Appendix Figure 3B** in **Supplementary Material**, **Table 3**).

#### 3.4.3 Depression

**Figure 4:**
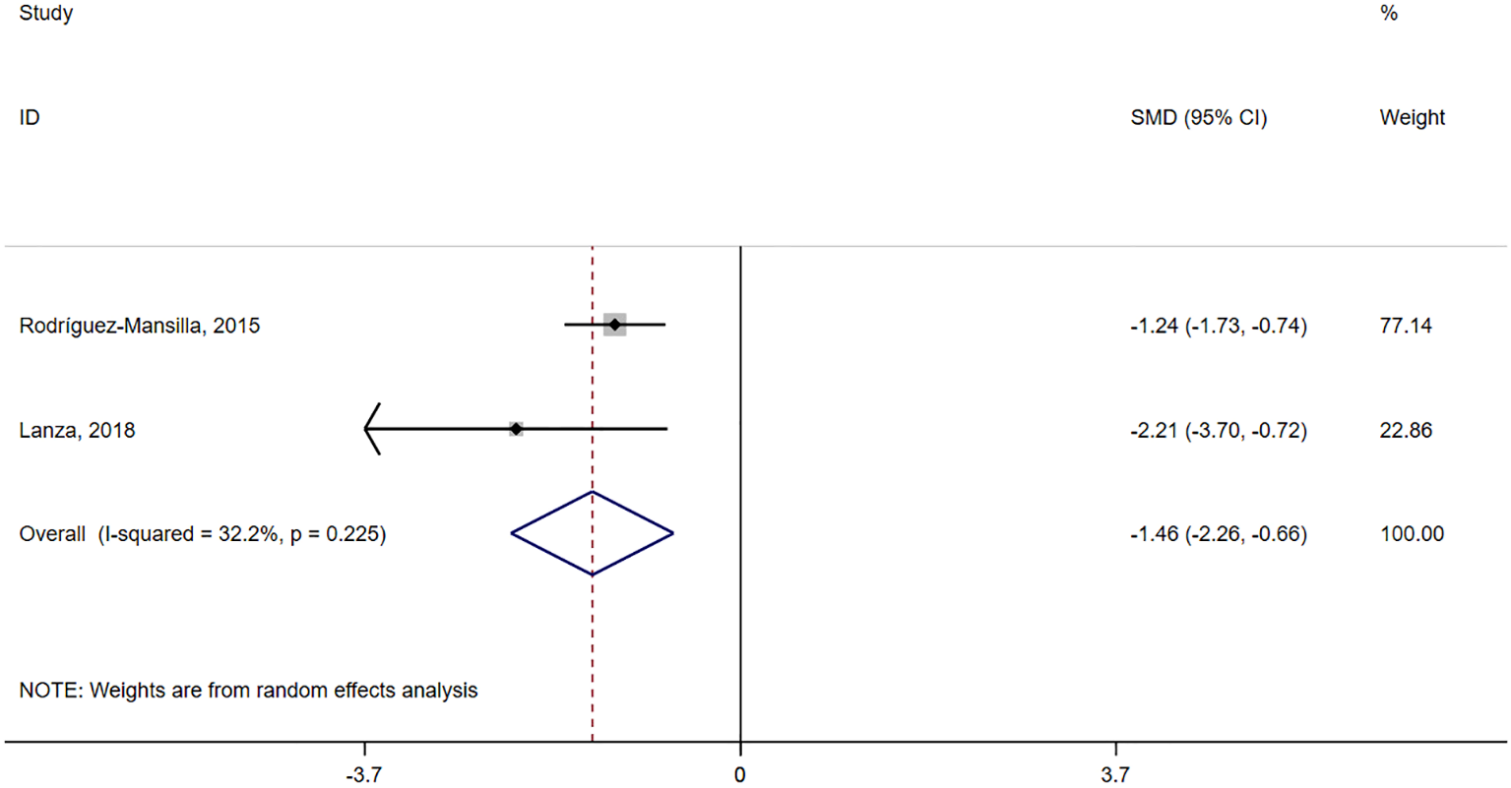
Forest plot of the effect of acupressure on depression.

Two studies (39, 40) reported on depression. One study (40) used the Cornell Scale for depression in dementia and the other (39) used the Geriatric Depression Scale. A statistically significant difference was seen between the two groups (SMD = -1.46, 95% confidence interval = -2.26 to -0.66, P<0.001, I² = 32.2%) (**Figure 4**, **Table 3**). The certainty of evidence for depression was low.

#### 3.4.4 ADL

**Figure 5:**
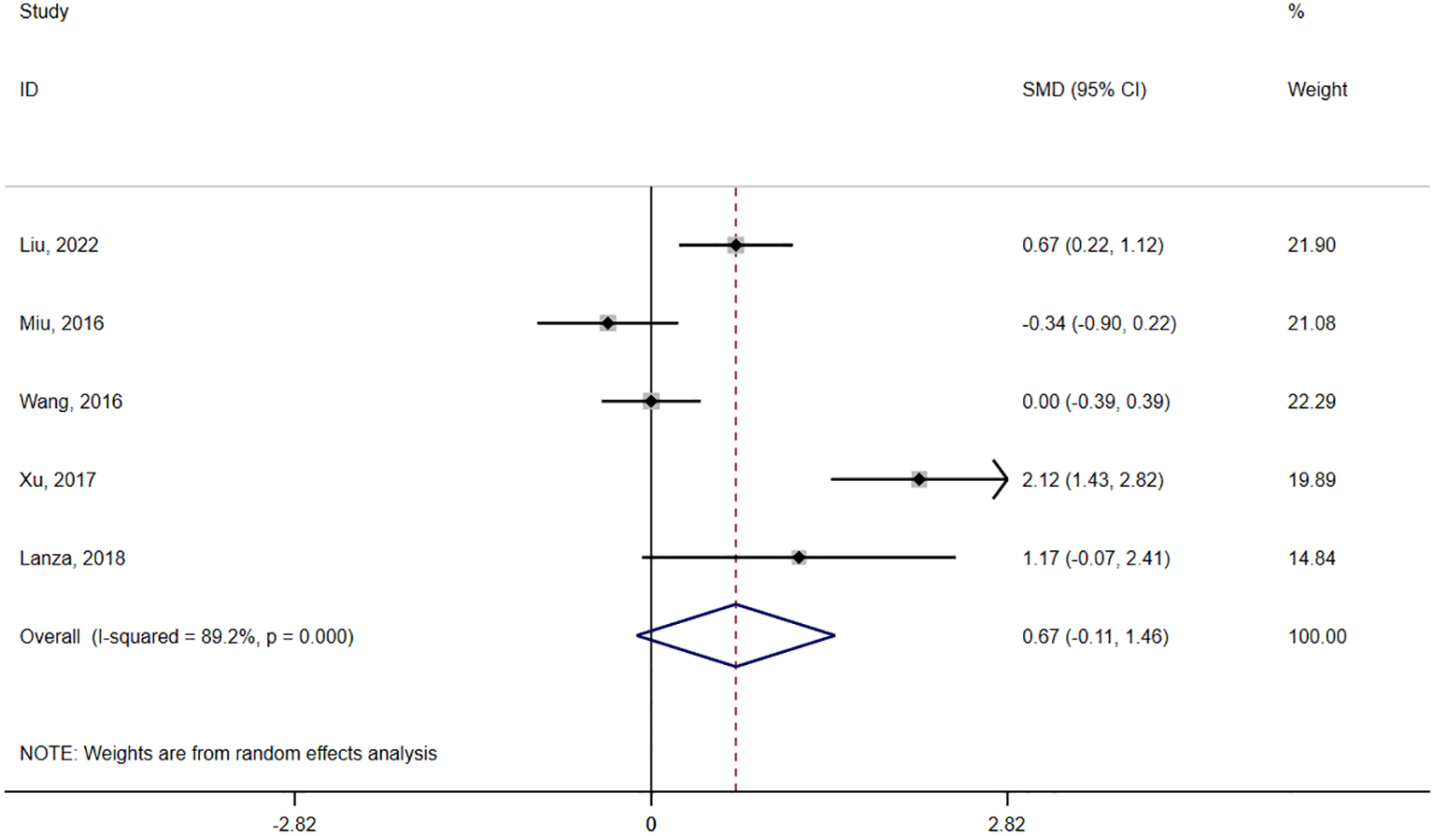
Forest plot of the effect of acupressure on ADL.

Five studies (29, 30, 32, 33, 39) reported on ADL. Three studies (29, 30, 39) used ADL scales, one (33) used the Functional Activities Questionnaire, and the other (32) used the Modified Barthel Index. No statistically significant difference in the ADL scores was observed between the groups (SMD = 0.67, 95% confidence interval = -0.11 to 1.46, P = 0.13, I² = 89.2%) (**Figure 5**, **Table 3**), and there was no evidence of publication bias (P for Egger’s regression test = 0.37) (**Appendix Table 1C** in **Supplementary Material**). By excluding Xu (29) in the sensitivity analysis, heterogeneity was reduced by 16%(SMD = 0.26, 95% confidence interval = -0.29 to 0.80, P = 0.36, I² = 73.2%) (**Appendix Table 2B** in **Supplementary Material**). The certainty of the evidence for the ADL was very low. Subgroup analysis was performed according to the intervention time, and the patients were divided into three subgroups: 5 months (29, 30), 6 months (32, 33), and 10 months (39) (**Appendix Figure 3C** in **Supplementary Material**, **Table 3**).

## 4 Discussion

To the best of our knowledge, this is the first meta-analysis to explore the effects of acupressure on cognition, mood, and ADL in older adults with CI. The results showed that acupressure can improve cognitive function and reduce agitation and depression in this demographic. However, a summary of existing data cannot prove that acupressure can improve the ADL of older people with CI. The Egger’s regression test showed no publication bias for any of the results. According to the PEDro scale assessment results, the methodological quality of all included studies was good and fair, and none were of poor quality. However, the GRADE score showed that the certainty of evidence for all results was low or very low, which resulted from the risk of bias, high heterogeneity, or insufficient sample size.

Physical stimulation through acupoint pressing can increase cerebral blood flow to the corresponding cortex, enhance blood oxygen and nutrient supply to brain tissue, regulate nervous system excitability, boost metabolism, improve immune function, and prevent or slow brain atrophy. This process initiates the body’s repair mechanisms, gradually restoring brain cells and enhancing cognitive function (42, 43). A functional magnetic resonance study showed that long-term acupressure modulates brain primary somatosensory activity and brain plasticity, which in turn affects sensory memory or learning (44). Results of an animal trial showed that auricular acupoint taping delayed learning and memory loss in rats, demonstrating that it can alleviate the effects of CI (45). This aligns with the collective outcomes reported in our analysis. Based on our meta-analysis and the support of animal experiments, we hope to conduct multicenter, large-sample RCTs in the future. High-quality RCTs have great value for clinical translation. Functional magnetic resonance imaging confirmed the specificity of acupoint stimulation points and revealed that stimulation may be associated with emotion regulation through activation of the amygdala and anterior cingulate cortex (46). Acupressure helps patients recover from psychogeriatric issues and general psychological distress (47). Acupressure massage can positively regulate neurotransmitter release, stimulating serotonin and beta-endorphin release, which helps the mood of older individuals (48–50). Serotonin is closely related to depression, which may be one of the mechanisms by which acupressure improves depression (51). Acupressure stimulation can engage the hypothalamic-pituitary-adrenal axis to counteract the overproduction of cortisol, which may be closely related to agitation in older adults (52, 53). This is consistent with our results revealing that acupressure improves depression and agitation in older people with CI. Acupressure mediates nitric oxide signaling, which is known to improve local microcirculation through cyclic guanosine monophosphate and helps improve physical performance by inhibiting fatigue-inducing molecules in the blood (54, 55). However, our pooled results showed that acupressure did not improve ADL in older people with CI. This may be because the pooled studies involved instrumental ADL, which requires patients to have a high level of cognitive function and processing ability, whereas acupressure can only improve the completion of basic ADL.

Because of the large heterogeneity in the overall results, we conducted subgroup analyses based on the intervention time. In the MMSE results, the effect size of the 6-month intervention was large, with lower heterogeneity compared to the overall results. However, the 10-month intervention yielded smaller effect sizes. We hypothesize that a 10-month intervention may be too prolonged, potentially leading to reduced patient adherence. Interventions that span long periods of time need to consider caregiver availability, frequent medical visits, and hospital admissions (39). The heterogeneity of other results still existed after subgroup analysis, which might have been caused by differences in research statistics or acupressure operations. Certain subgroups included only one study, and the effectiveness of the subgroups could not be verified. Therefore, more studies are needed to verify the reliability of the evidence of acupressure in terms of intervention time. Other than that, acupressure combined with cognitive therapy did not show an advantage in terms of cognitive improvement. The high heterogeneity of results was due to the fact that Guo et al. (41) intervened for only 6 weeks, while Wang (30) intervened for 6 months. Therefore, the results would have shown conservative under random effects model fitting. The heterogeneity of the MMSE results decreased after sensitivity analysis, and the effect size did not change much. Therefore, the results of acupressure improving cognitive function are relatively reliable. There are no standardized acupressure points for the treatment of CI, so there were differences in the selection of acupressure points included in the study. There were also differences in the different non-acupuncture point interventions in the control group. These may be factors for increased heterogeneity. In addition to these, patients with CI vary in severity and sensitivity to acupressure, which may also lead to increased heterogeneity. Further exploration of these factors is necessary in the future. In this study, the order of the most used acupoints were Baihui(GV20), Fengchi(GB20), and Taiyang(EX-HN5). In clinical practice, these points may be prioritized. In terms of intervention duration, a 6-month intervention may be more likely to showed advantages in improving cognition. This is due to the fact that the effect size of six months (MD = 2.66) is greater than the other intervention durations.

Acupressure, an acupuncture technique, has many advantages in clinical treatment and is worth promoting further. Acupressure and acupuncture are based on the same therapeutic principle of activating acupoints through meridians (10, 56). However, acupressure is noninvasive and safe. For patients who are not comfortable with needles during their first encounter, acupressure can be used as a transitional treatment. Acupressure can be self-administered and is compatible with drug therapy (57). The treatment is simple and can be performed by oneself after professional training, which is convenient for patients. Self-treatment greatly reduces the cost of treatment and personnel costs, improves the efficiency of treatment, and reduces medical pressure on society.

Our study has certain limitations. First, the number of included RCTs and study participants was small, which affected the power of the results. In addition, the certainty of evidence for the results of this study was “Low” and “Very Low”. This may impact the robustness of the conclusions. Therefore, these results should be interpreted with caution. Second, there is currently no guideline for acupoint combinations for acupressure, and it is impossible to compare the therapeutic effects of different acupoint combinations in the treatment of older people with CI. Third, the MMSE only represents overall cognitive function, and the effect of acupressure on functions in the sub-cognitive domains is unclear. Finally, most studies did not conduct follow-up surveys. Our study was unable to explore the continuity of the therapeutic effect of acupressure based on follow-up data from the original study. Future research on acupressure needs to clarify this aspect and further quantify the intervention parameters to improve treatment efficiency. Despite extensive reviews summarizing evidence-based medical data demonstrating the beneficial effects of acupressure on cognition and mood in older adults with CI, many uncertainties remain.

Our study delineates four critical avenues for future research. First, multicenter randomized controlled trials should be conducted with subgroup analyses stratified by distinct cognitive stages or acupoint pairings. Second, mechanistic investigations in animal models should employ neuroimaging modalities such as functional near-infrared spectroscopy and functional magnetic resonance imaging to elucidate acupressure’s biomolecular pathways and central nervous system interactions. Third, the development of intelligent acupressure systems integrating pressure sensors with electroencephalogram feedback could enable dynamic optimization of personalized treatment protocols. Finally, systematic evaluation of acupressure as an adjunct to cognitive rehabilitation programs may help elucidate potential synergistic effects, thereby informing the development of comprehensive multimodal intervention strategies.

## 5 Conclusion

This study used a systematic review and meta-analysis method to explore the effects of acupressure on cognition, mood, and ADL in older adults with CI. The results showed that acupressure can improve cognitive function, agitation, and depression in this population. However, there was no statistically significant difference in ADL between the experimental and control groups. Future studies should investigate the effects of different acupoint combinations.

## Data Availability

The original contributions presented in the study are included in the article/supplementary material. Further inquiries can be directed to the corresponding author.
