# Supplementary material for: Acupressure for older people with cognitive impairment: a systematic review and meta-analysis of randomized controlled trials
Source: Front Psychiatry. 2025 Apr 25;16:1548878. doi: 10.3389/fpsyt.2025.1548878 (PMC12062119; doi:10.3389/fpsyt.2025.1548878)
Supplement: Supplementary file 1 [file SupplementaryFile1.docx]

**Appendix**

**eTable1.** PRISMA 2009 checklist

**eTable2.** Search strategy from database inception to March 16, 2025

**eTable3.** Studies excluded from all randomized controlled trials after reading the full text and reasons for exclusion

**eTable4**. The results of GRADE assessment of the evidence certainty

**eFigure1.** Egger’s regression test of results

**eFigure2.** Sensitivity analysis of results

**eFigure3.** Subgroup analysis of results

**eTable1.** PRISMA 2009 checklist

| **Section/topic** | **#** | **Checklist item** | **Reported on page #** |
| --- | --- | --- | --- |
| **TITLE** | | |  |
| Title | 1 | Identify the report as a systematic review, meta-analysis, or both. | 1 |
| **ABSTRACT** | | |  |
| Structured summary | 2 | Provide a structured summary including, as applicable: background; objectives; data sources; study eligibility criteria, participants, and interventions; study appraisal and synthesis methods; results; limitations; conclusions and implications of key findings; systematic review registration number. | 2-3 |
| **INTRODUCTION** | | |  |
| Rationale | 3 | Describe the rationale for the review in the context of what is already known. | 4-5 |
| Objectives | 4 | Provide an explicit statement of questions being addressed with reference to participants, interventions, comparisons, outcomes, and study design (PICOS). | 4-5 |
| **METHODS** | | |  |
| Protocol and registration | 5 | Indicate if a review protocol exists, if and where it can be accessed (e.g., Web address), and, if available, provide registration information including registration number. | 5-6 |
| Eligibility criteria | 6 | Specify study characteristics (e.g., PICOS, length of follow-up) and report characteristics (e.g., years considered, language, publication status) used as criteria for eligibility, giving rationale. | 6-7 |
| Information sources | 7 | Describe all information sources (e.g., databases with dates of coverage, contact with study authors to identify additional studies) in the search and date last searched. | 6 |
| Search | 8 | Present full electronic search strategy for at least one database, including any limits used, such that it could be repeated. | 6 |
| Study selection | 9 | State the process for selecting studies (i.e., screening, eligibility, included in systematic review, and, if applicable, included in the meta-analysis). | 6 |
| Data collection process | 10 | Describe method of data extraction from reports (e.g., piloted forms, independently, in duplicate) and any processes for obtaining and confirming data from investigators. | 7 |
| Data items | 11 | List and define all variables for which data were sought (e.g., PICOS, funding sources) and any assumptions and simplifications made. | 7 |
| Risk of bias in individual studies | 12 | Describe methods used for assessing risk of bias of individual studies (including specification of whether this was done at the study or outcome level), and how this information is to be used in any data synthesis. | 7-8 |
| Summary measures | 13 | State the principal summary measures (e.g., risk ratio, difference in means). | 8 |
| Synthesis of results | 14 | Describe the methods of handling data and combining results of studies, if done, including measures of consistency (e.g., I^2^) for each meta-analysis. | 8-9 |
| Risk of bias across studies | 15 | Specify any assessment of risk of bias that may affect the cumulative evidence (e.g., publication bias, selective reporting within studies). | 8-9 |
| Additional analyses | 16 | Describe methods of additional analyses (e.g., sensitivity or subgroup analyses, meta-regression), if done, indicating which were pre-specified. | 8-9 |

| **Section/topic** | **#** | **Checklist item** | **Reported on page #** |
| --- | --- | --- | --- |

| **RESULTS** | | |  |
| --- | --- | --- | --- |
| Study selection | 17 | Give numbers of studies screened, assessed for eligibility, and included in the review, with reasons for exclusions at each stage, ideally with a flow diagram. | 9 |
| Study characteristics | 18 | For each study, present characteristics for which data were extracted (e.g., study size, PICOS, follow-up period) and provide the citations. | 9-10 |
| Risk of bias within studies | 19 | Present data on risk of bias of each study and, if available, any outcome level assessment (see item 12). | 10 |
| Results of individual studies | 20 | For all outcomes considered (benefits or harms), present, for each study: (a) simple summary data for each intervention group (b) effect estimates and confidence intervals, ideally with a forest plot. | 10-12 |
| Synthesis of results | 21 | Present results of each meta-analysis done, including confidence intervals and measures of consistency. | 10-12 |
| Risk of bias across studies | 22 | Present results of any assessment of risk of bias across studies (see Item 15). | 10 |
| Additional analysis | 23 | Give results of additional analyses, if done (e.g., sensitivity or subgroup analyses, meta-regression [see Item 16]). | 10-12 |
| **DISCUSSION** | | |  |
| Summary of evidence | 24 | Summarize the main findings including the strength of evidence for each main outcome; consider their relevance to key groups (e.g., healthcare providers, users, and policy makers). | 12-13 |
| Limitations | 25 | Discuss limitations at study and outcome level (e.g., risk of bias), and at review-level (e.g., incomplete retrieval of identified research, reporting bias). | 15 |
| Conclusions | 26 | Provide a general interpretation of the results in the context of other evidence, and implications for future research. | 16 |
| **FUNDING** | | |  |
| Funding | 27 | Describe sources of funding for the systematic review and other support (e.g., supply of data); role of funders for the systematic review. | 17 |

From: Moher, D., Liberati, A., Tetzlaff, J., Altman, D. G., & PRISMA Group. Preferred reporting items for systematic reviews and meta-analyses:

the PRISMA statement. Ann Intern Med 151, 264–269, W64 (2009).

**eTable2.** Search strategy from database inception to June 6, 2024

| **Database** | **Search term** | **Results** |
| --- | --- | --- |
| PubMed | (((((acupressure[Title/Abstract]) OR (Shiatsu[Title/Abstract])) OR (Zhi Ya[Title/Abstract])) OR (Chih Ya[Title/Abstract])) AND ((((((cogni*[Title/Abstract]) OR (cognitive decline*[Title/Abstract])) OR (cognitive fail*[Title/Abstract])) OR (age-associated memory impairment[Title/Abstract])) OR (alzheimer*[Title/Abstract])) OR (dement*[Title/Abstract]))) AND (((randomized controlled trial*[Title/Abstract]) OR (randomized controlled studies[Title/Abstract])) OR (randomized controlled experiments[Title/Abstract])) | 32 |
| Embase | ('acupressure'/exp/mj OR shiatsu:ab,ti OR 'zhi ya':ab,ti OR 'chih ya':ab,ti) AND ('cognition'/exp/mj OR cogni*:ab,ti OR 'cognitive decline*':ab,ti OR 'cognitive fail*':ab,ti OR 'age-associated memory impairment':ab,ti OR alzheimer*:ab,ti OR dement*:ab,ti) AND ('randomized controlled trial'/exp/mj OR 'randomized controlled trial*':ab,ti OR 'randomized controlled studies':ab,ti OR 'randomized controlled experiments':ab,ti) | 22 |
| Cochrane Library | ((acupressure):ti,ab,kw OR (Shiatsu):ti,ab,kw OR (Zhi Ya):ti,ab,kw OR (Chih Ya):ti,ab,kw) AND ((cogni*):ti,ab,kw OR (cognitive decline*):ti,ab,kw OR (cognitive fail*):ti,ab,kw OR (age-associated memory impairment):ti,ab,kw OR (alzheimer*):ti,ab,kw OR (dement*):ti,ab,kw ) AND ((randomized controlled trial*):ti,ab,kw OR (randomized controlled studies):ti,ab,kw OR (randomized controlled experiments):ti,ab,kw) | 57 |
| Web of Science | (acupressure (Title) or acupressure (Abstract) or Shiatsu (Title) or Shiatsu (Abstract) or Zhi Ya (Title) or Zhi Ya (Abstract) or Chih Ya (Title) or Chih Ya (Abstract)) AND (cogni* (Title) or cogni* (Abstract) or cognitive decline* (Title) or cognitive decline* (Abstract) or cognitive fail* (Title) or cognitive fail* (Abstract) or age-associated memory impairment (Title) or age-associated memory impairment (Abstract) or alzheimer* (Title) or alzheimer* (Abstract) or dement* (Title) or dement* (Abstract)) AND (randomized controlled trial* (Title) or randomized controlled trial* (Abstract) or randomized controlled studies (Title) or randomized controlled studies (Abstract) or randomized controlled experiments (Title) or randomized controlled experiments (Abstract)) | 48 |
| Sinomed | ("穴位按压"[常用字段:智能] OR "穴位按摩"[常用字段:智能] OR "指压"[常用字段:智能]) AND ("认知功能障碍"[常用字段:智能] OR "阿尔兹海默症"[常用字段:智能] OR "痴呆"[常用字段:智能]) | 60 |
| CNKI | (主题: 穴位按压) OR (篇关摘: 穴位按压 + 穴位按摩 + 指压(精确)) AND (主题: 认知功能障碍) OR (篇关摘: 认知功能障碍 + 痴呆 + 阿尔兹海默症 (精确)) | 29 |
| Wanfang | 题名或关键词:(穴位按压 or 穴位按摩 or 指压) and 题名或关键词:(认知功能障碍 or 阿尔兹海默症 or 痴呆) | 42 |
| VIP | (题名或关键词=穴位按摩 or 穴位按压 or 指压) AND (题名或关键词=认知功能障碍 or 痴呆 or 阿尔兹海默症) | 32 |
| **Total** |  | 322 |

**eTable3.** Studies excluded from all randomized controlled trials after reading the full text and reasons for exclusion

| Not RCTs (n=9) | 1. Zeng Q. Application of head acupoint massage and geriatric specialist nursing in the care of Alzheimer's patients. *Health must-Read magazine*. 2018;(23):180. 2. Chen XL, Wang YP. Acupoint massage for constipation in 50 patients with senile dementia. *Zhejiang Journal of Traditional Chinese Medicine*. 2009,44(2):138. doi:10.3969/j.issn.0411-8421.2009.02.043. 3. Feng XM, Wang SH. Effect of acupoint massage on cognitive function in elderly patients with mild cognitive impairment. *Chinese Journal of Gerontology*. 2015(18):5257-5259. doi:10.3969/j.issn.1005-9202.2015.18.106. 4. Huang L. Clinical Observation on Acupoint Massage in Treating Senile Dementia. *Massage and Guidance*. 1995, (11):25-26. 5. Meng L. Acupoint massage for the treatment of Alzheimer's disease. *Massage and Guidance*. 2000, (3):24-49. 6. Qian ML. A comparative study on the clinical effectiveness of two nursing intervention models for patients with mild cognitive impairment. *China's primary care medicine.* 2015(14):2235-2236,2237. doi:10.3760/cma.j.issn.1008-6706.2015.14.053. 7. Wan CX, Hu ZF, Sun YQ, Yang XL. Application of acupoint massage in nursing care of community dementia patients. *Nursing Research*. 2017,31(28):3524-3527. doi:10.3969/j.issn.1009-6493.2017.28.011. 8. Wei SF, Song HX. Clinical observation of physical therapy combined with intensive nursing intervention for Alzheimer's disease. *Journal of Clinical Rational Drug Use.* 2011,4(25):90-91. doi:10.3969/j.issn.1674-3296.2011.25.073. 9. Zhang DY, Wang SL, Liao Z. High-voltage electronic pen acupuncture for the treatment of senile dementia. *Chinese Clinical Rehabilitation*. 2002,6(17):2622-2623. doi:10.3321/j.issn:1673-8225.2002.17.114. |
| --- | --- |
| Incorrect population (n=2) | 1. Cheung DST, Tiwari A, Yeung WF, et al. Self-Administered Acupressure for Caregivers of Older Family Members: A Randomized Controlled Trial. Article. *Journal of the American Geriatrics Society.* 2020;68(6):1193-1201. doi:10.1111/jgs.16357 2. Ho FYY, Choi WT, Yeung WF, Lam HK, Lau WY, Chung KF. The efficacy of integrated cognitive behavioral therapy (CBT) and acupressure versus CBT for insomnia: a three-arm pilot randomized controlled trial. Article. *Sleep Medicine.* 2021;87:158-167. doi:10.1016/j.sleep.2021.08.024 |
| Not relevant results (n=3) | 1. Bayram S, Altınbaş Akkaş Ö, Usta E. Effect of acupressure on agitation in the elderly with dementia who receive institutional care: a pilot study. Journal article. Perspectives in psychiatric care. 2021;57(3):980‐988. doi:10.1111/ppc.12642 2. Zhang JJ, Yu L, Mei JH, et al. Effect of Auricular Acupressure on Acute Pain in Nursing Home Residents with Mild Dementia: A Single-Blind, Randomized, Sham-Controlled Study. *Evidence-Based Complementary and Alternative Medicine.* Mar 2022;20226406383. doi:10.1155/2022/6406383 3. Ovliaei Bidgoli M, Tagharrobi Z, Sharifi K, Sooki Z, Momen-Heravi M, Zare Joshaghani F, Zare M. The effect of ear acupressure on occupational cognitive failure in nurses after the COVID-19 crisis: a randomized controlled clinical trial. *BMC Nurs*. 2024 Jul 5;23(1):457. doi: 10.1186/s12912-024-02139-w. |
| Unavailable data (n=2) | 1. Zeng H, Liu M, Wang P, Kang J, Lu F, Pan L. The Effects of Acupressure Training on Sleep Quality and Cognitive Function of Older Adults: a 1-Year Randomized Controlled Trial. Journal article. *Research in nursing & health.* 2016;39(5):328‐336. doi:10.1002/nur.21738 2. Wang Y, Kui JZ. Application of acupoint massage in nursing care of patients with senile dementia. *Yunnan Journal of Traditional Chinese Medicine*. 2014,35(4):81-82. |
| Unavailable full text (n=2) | 1. Kwan RYC, Leung MCP, Lai CKY. Effects of acupressure on agitation and stress in nursing home residents with dementia. Journal article; Conference proceeding. *Alzheimer's & dementia.* 2017;13(7):P931. 2. Chi ZJ. Effects of ear acupoint seed embedding combined with acupoint massage on cognitive function and daily living ability in patients with Alzheimer's disease. *Women's Health.* 2023;(23):199-200. |

**eTable4**. The results of GRADE assessment of the evidence certainty

| Outcomes | Study Design | Risk of Bias | Inconsistency | Indirectness | Imprecision | Publication bias | Other Consideration | Numbers of Participants | | Metric | Absolute Effect (95% confidence interval) | Certainty |
| --- | --- | --- | --- | --- | --- | --- | --- | --- | --- | --- | --- | --- |
|  |  |  |  |  |  |  |  | EG | CG |  |  |  |
| Cognition | RCTs | serious ^a^ | no | no | serious ^b^ | no | no | 151 | 149 | MD | 2.36 (1.71, 3.00) | Low |
| Agitation | RCTs | serious ^a^ | no | no | serious ^b^ | no | no | 169 | 195 | MD | -1.51 (-2.52, -0.50) | Low |
| Depression | RCTs | serious ^a^ | no | no | serious ^b^ | no | no | 46 | 41 | SMD | -1.33 (-1.80, -0.86) | Low |
| ADL | RCTs | serious ^a^ | serious ^c^ | no | serious ^b^ | no | no | 146 | 146 | SMD | 0.26 (-0.29, 0.80) | Very low |

**Abbreviation:** GRADE: Grading of Recommendations Assessment, Development and Evaluation EG: experimental group; CG: control group; RCTs: randomized controlled trials; ADL: activities of living.

**Criteria:** ^a^ Downgraded by one level for the risk of bias: If 1 or more of the 3 criteria (randomization, masking, dropout rate ≤30%) is not met in 10-30% of trials included in the systematic review.

^b^ Downgraded by one level for imprecision: Continuous variable events < 400.

^c^ Downgraded by one level for inconsistency: Substantial heterogeneity is seen between studies (I²＞50%).

^d^ Downgraded by one level for indirectness: Studies in different contexts have participants, interventions, or results that are not consistent with the actual question.

^e^ Downgraded by one level for publication bias: In Egger’s test, P -values < 0.05.

**eTable5**. Description of the scales.

| Scales and Tests | Interpretations |
| --- | --- |
| MMSE(1) | The MMSE is categorized into Orientation, Memory, Attention and Numeracy, Recall, and Language. There are 30 questions in total, and the scores for each question are added together for a total of 30 points. The lower the score, the more severe the cognitive impairment may be. It is mainly used to rapidly assess the degree of cognitive impairment in adults, and is especially widely used in the clinical diagnosis and follow-up of dementia (e.g., Alzheimer's disease), delirium, and other diseases. |
| MoCA(2) | The MoCA is designed for the early identification of mild cognitive impairment (MCI) and neurodegenerative diseases (e.g., Alzheimer's disease, Parkinson's disease dementia). The scale assesses 8 major cognitive domains (including visuospatial/executive functioning, attention, delayed recall, abstract thinking, etc.) through 12 tasks with a total score of 30. Its core advantage is that it can effectively capture frontal and executive function impairments that are easily overlooked by conventional scales (e.g., MMSE), and it is especially suitable for early screening of people with higher education levels. |
| CAMI(3) | The CAMI is used to assess agitated behavior in patients with dementia. It contains 29 entries assessing the frequency and type of agitated behavior, such as physical aggression, verbal aggression, and non-aggressive behavior. Each behavior is rated according to the frequency of occurrence, usually from 1 (never) to 7 (several times per hour). |
| GDS(4) | The GDS is a self-assessment screening tool specifically designed to assess depressive symptoms in older adults over the age of 60, with the core strength of excluding somatic symptoms (e.g., fatigue, insomnia) and focusing on emotional and cognitive dimensions (e.g., feelings of hopelessness, life satisfaction). The scale consists of either a 30-item (long version) or a 15-item (short version) short question and answer (yes/no) dichotomous scale, with higher total scores increasing the risk of depression (GDS-15 cut-off score ≥5 suggests possible depression). It is particularly suitable for older adults with comorbid chronic conditions or mild cognitive impairment, with the caveat that those with severe dementia or language impairment need to be assisted in completing it by a caregiver. |
| BI(5) | The Barthel Index is a specially designed assessment tool for activities of daily living (ADL), which quantifies the patient's level of independent functioning through 10 basic activities (e.g., eating, bathing, dressing, toileting, walking, etc.), with a score of 0-15 points for each item, and a total score range of 0-100 points (the higher the score, the greater the independence). |
| FAQ(6) | The FAQ scale is a tool for assessing ability to perform instrumental activities of daily living (ADLs), focusing on independence in complex life tasks (e.g., money management, shopping, medication management, transportation, etc.). The scale consists of 10 activities that are rated by the patient or informant on the basis of ability to complete them (0=completely independent, 3=completely dependent), with a total score of 0-30 (≥6 indicates functional impairment). Its core strength is its sensitivity to capture early cognitive decline or mild functional impairment (e.g., prodromal Alzheimer's disease). |

MMSE: Mini-Mental State Examination; MoCA: Montreal Cognitive Assessment; CAMI: Cohen-Mansfield Agitation Inventory; GDS: Geriatric Depression Scale;

BI: Barthel index; FAQ: Functional Activities Questionnaire.


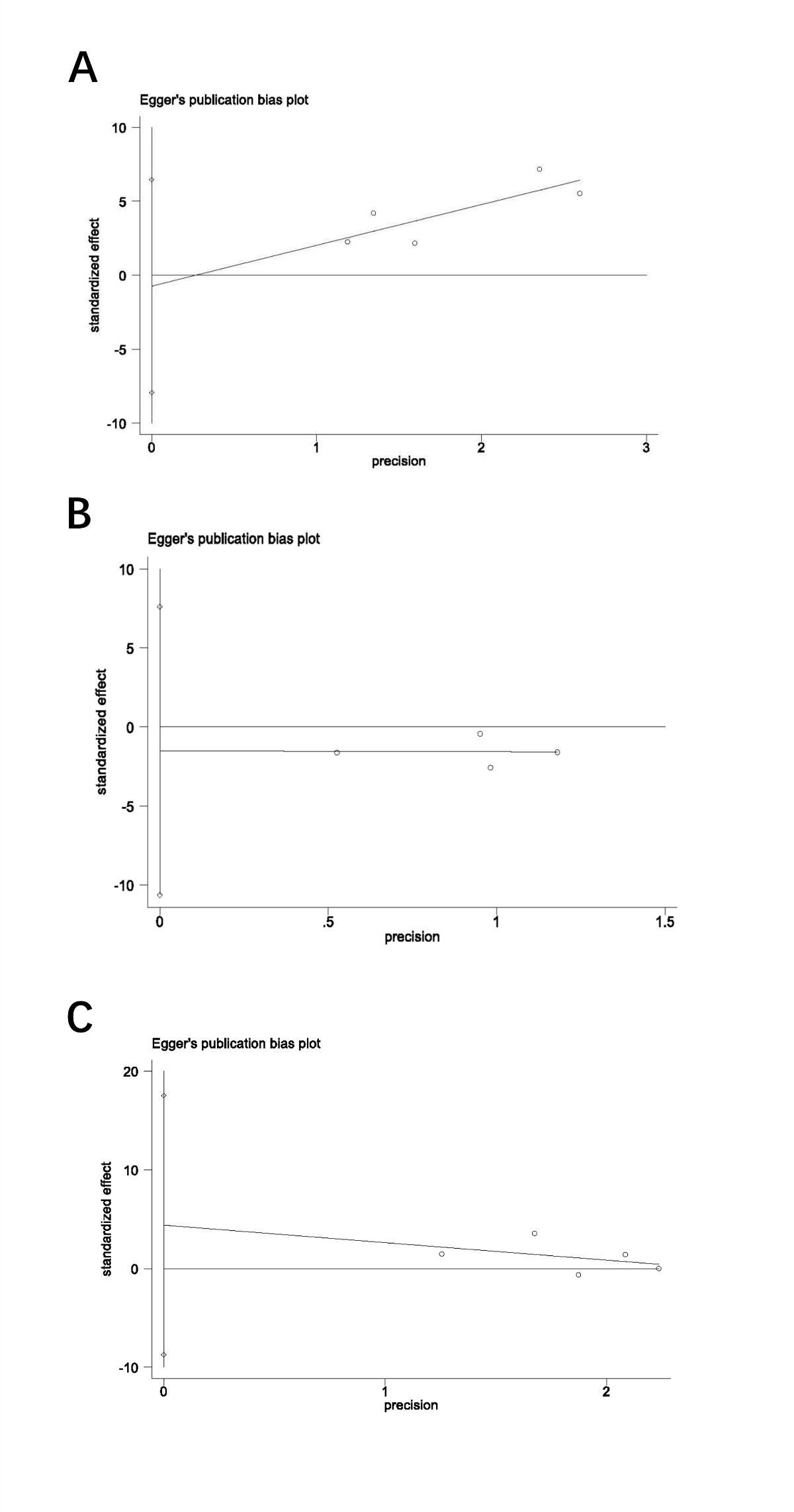


**eFigure1.** Egger’s regression test of results: A. Cognition; B. Agitation; C. Activities of living (ADL)


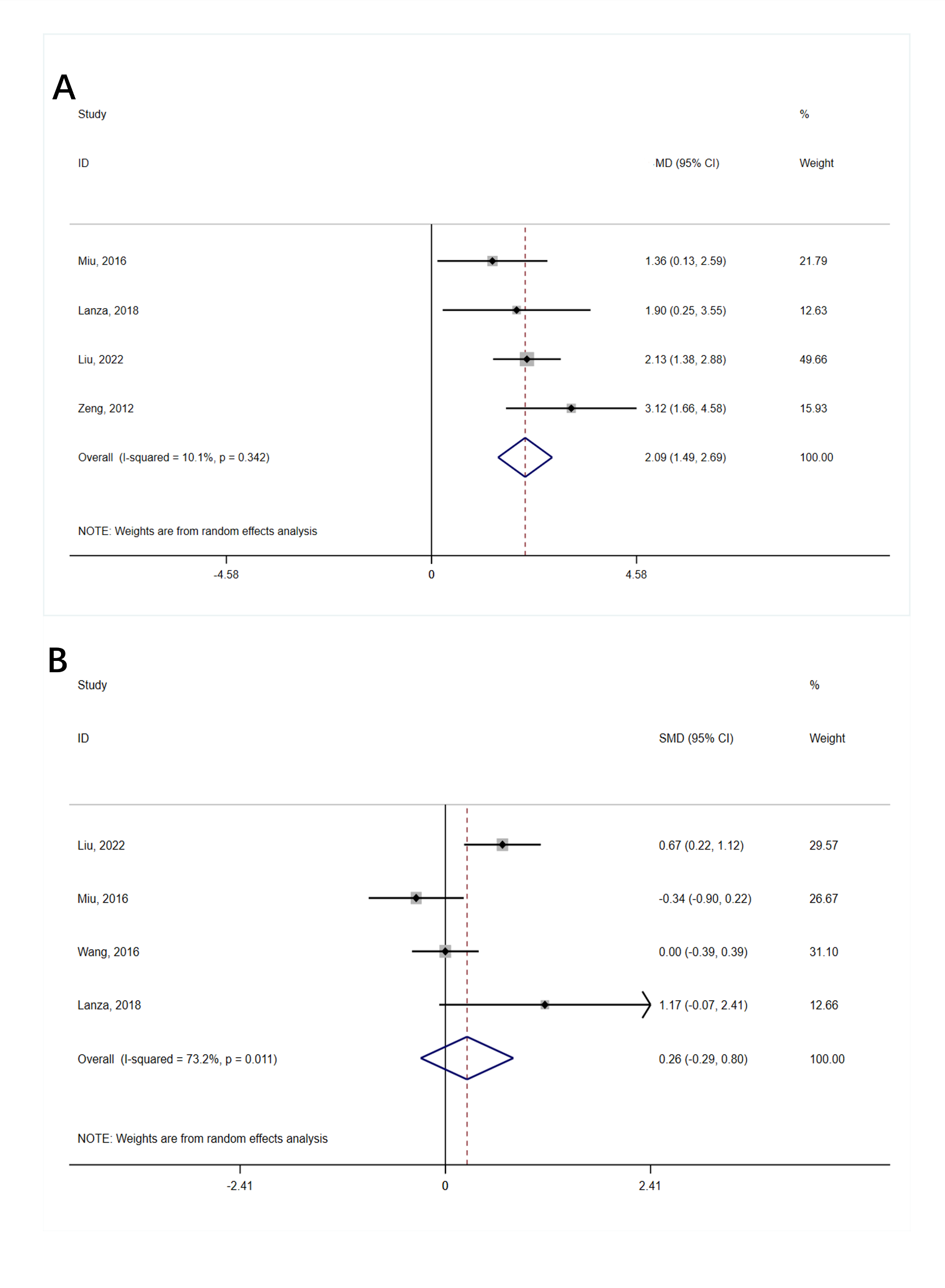


**eFigure2.** Sensitivity analysis of results: A. Cognition; B. Activities of living (ADL)


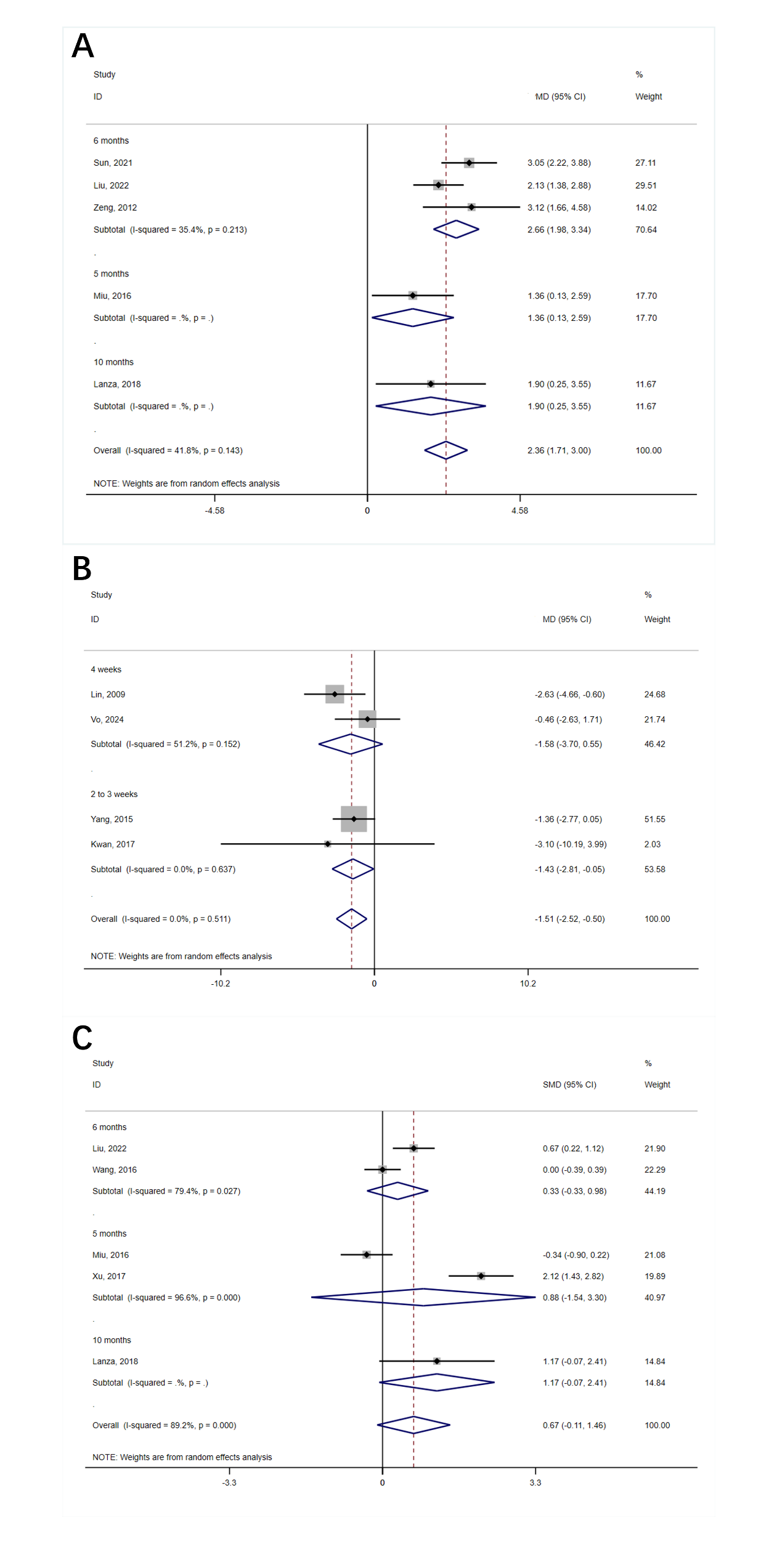


**eFigure3.** Subgroup analysis of results: A. Cognition; B. Agitation; C. Activities of living.
